# Cocreation of Massive Open Online Courses to Improve Digital Health Literacy in Diabetes: Pilot Mixed Methods Study

**DOI:** 10.2196/30603

**Published:** 2021-12-13

**Authors:** Yolanda Alvarez-Perez, Lilisbeth Perestelo-Perez, Amado Rivero-Santana, Ana M Wagner, Alezandra Torres-Castaño, Ana Toledo-Chávarri, Andrea Duarte-Díaz, Dácil Alvarado-Martel, Barbara Piccini, Stephan Van den Broucke, Jessica Vandenbosch, Carina González-González, Michelle Perello, Pedro Serrano-Aguilar, IC-Health Project Consortium

**Affiliations:** 1 Canary Islands Health Research Institute Foundation (FIISC) Tenerife Spain; 2 Evaluation Unit (SESCS) Canary Islands Health Service (SCS) Tenerife Spain; 3 Health Services Research on Chronic Patients Network (REDISSEC) Tenerife Spain; 4 Center for Biomedical Research of the Canary Islands (CIBICAN) Tenerife Spain; 5 Department of Endocrinology and Nutrition University Hospital Insular Materno-Infantil Las Palmas de Gran Canaria Spain; 6 Research Institute of Biomedical and Health Sciences University of Las Palmas de Gran Canaria Las Palmas de Gran Canaria Spain; 7 Diabetology Unit Meyer University Children’s Hospital Florence Italy; 8 Université Catholique de Louvain Louvain Belgium; 9 Interaction, Technology, and Education Research Group Department of Computer Science and Engineering University of La Laguna La Laguna Spain; 10 Consulta Europa Projects and Innovation Las Palmas de Gran Canaria Spain; 11 See Acknowledgements

**Keywords:** diabetes, digital health literacy, health education, MOOC

## Abstract

**Background:**

Self-management education is a fundamental aspect in the health care of people with diabetes to develop the necessary skills for the improvement of health outcomes. Patients are required to have the competencies to manage electronic information resources—that is, an appropriate level of digital health literacy. The European project IC-Health aimed to improve digital health literacy among people with diabetes through the cocreation of massive open online courses (MOOCs).

**Objective:**

We report the preliminary results obtained in 3 participating countries in the IC-Health project (Italy, Spain, and Sweden) regarding (1) experience of the participants during the cocreation process of MOOCs, (2) perceived changes in their digital health literacy level after using MOOCs, and (3) a preliminary assessment of the acceptability of MOOCs.

**Methods:**

The cocreation of the MOOCs included focus groups with adults and adolescents with diabetes and the creation of independent communities of practice for type 1 diabetes and type 2 diabetes participants aimed to co-design the MOOCs. Quantitative measures of the acceptability of MOOCs, experience in the cocreation process, and increase in digital health literacy (dimensions of finding, understanding, and appraisal) were assessed.

**Results:**

A total of 28 participants with diabetes participated in focus groups. Adults and adolescents agreed that the internet is a secondary source of health-related information. A total of 149 participants comprised the diabetes communities of practice. A total of 9 MOOCs were developed. Acceptability of the MOOCs and the cocreation experience were positively valued. There was a significant improvement in digital health literacy in both adults and adolescents after using MOOCs (*P*<.001).

**Conclusions:**

Although the results presented on self-perceived digital health literacy are preliminary and exploratory, this pilot study suggests that IC-Health MOOCs represent a promising tool for the medical care of diabetes, being able to help reduce the limitations associated with low digital health literacy and other communication barriers in the diabetes population.

## Introduction

### Background

Diabetes is a chronic disease leading to severe morbidity, reduced quality of life, and anticipated mortality. According to the Diabetes Atlas of the International Diabetes Federation, more than 59 million adults aged 20 to 79 years in the European Union had diabetes in 2019 and it is estimated to reach 68 million in 2045 [1].

Self-management education is a fundamental aspect in the health care of people with diabetes to increase knowledge about their disease and develop the necessary skills to improve glycemic control and health outcomes [2]. Structured education programs have proven to be cost-effective to improve glycemic control and patient quality of life and reduce diabetes complications [3]. However, not all people with diabetes have access to these interventions due to financial barriers or limited offer by the health care system, among others [4,5].

These limitations in glucose control can be partly overcome through technological advances such as continuous glucose monitoring systems or insulin pumps. The daily use of these medical devices has improved the quality of life of people with diabetes [6] and requires some degree of health literacy [7,8] or digital health literacy [9,10]. The skills related to digital health literacy are to find, understand, appraise, and apply health information from electronic sources and apply the knowledge gained to addressing or solving a health problem [11]. Several studies have shown that internet-based diabetes education may improve patient knowledge and ability to access and interpret online health information, provide greater interaction with health care professionals, and promote better self-management of health conditions, healthier lifestyles, diabetes control, and quality of life [12-17]. Involvement in online peer support communities can be a beneficial adjunct to learning, serving as an option for ongoing diabetes peer support [18,19]. However, a barrier to the use of internet may be a lack of knowledge about how to find and interpret information online, since having access to technology is not necessarily associated with knowing how to use it [20,21].

Massive open online courses (MOOCs), a type of open educational resource [22], are innovative tools to improve education and practice, easily applicable to empower patients with chronic conditions to find quality, equitable, patient-centered education aimed at better health outcomes [23-25]. Cocreation is an option to enhance the relevance and usability of MOOCs by involving potential users and health care professionals, resulting in an effective strategy to design possible solutions aimed at increasing self-efficacy and empowerment of patients [26-29].

The European Commission works on the development of specific health innovation initiatives aimed to empower patients and promote the adoption of eHealth across the European Union, as can be seen in some programs and plans [30].

In this regard, the European project IC-Health: Improving Digital Health Literacy in Europe aimed to improve the digital health literacy level of European people with diabetes and other population cohorts through the cocreation of MOOCs focusing on the essential digital health literacy skills [31].

### Objectives

This study aimed to develop MOOCs designed to improve the digital health literacy level of people with type 1 diabetes (T1D) and type 2 diabetes (T2D) in 5 European countries (Spain, Belgium, Denmark, Italy, and Sweden) under the framework of the IC-Health project. In this paper, we present (1) the results of the focus groups run to explore the experience of people with diabetes in the use of the internet for health-related issues, as well as their needs and expectations, in order to inform the MOOCs’ development; (2) the cocreation methodology applied and the developed MOOCs; and (3) a pilot assessment of participant experiences in the cocreation process, the acceptability of the MOOCs, and their effect on self-perceived digital health literacy.

## Methods

### Ethics

The partner organizations were responsible for processing the necessary procedures to request approval by the corresponding ethical committees to evaluate their organization, and they assured the compatibility of the research activities with national and European ethics requirements in order to protect the rights, safety, and well-being of participants involved. An internal ethical committee was created comprising representatives appointed by each project partner and identified among highly skilled professional experts in any of the following areas: public health, health care evaluation, health promotion, social research, engineering, development, or human rights. The presence of different national members ensured that any country-specific ethical requirements were considered throughout the project life. Partners required approvals from the internal ethical committee to perform cocreation activities for the project.

### Study Design

A broader description of the design and methodology of the IC-Health project can be found in Perestelo-Pérez et al [32]. It included a review of the literature, exploratory survey with T1D and T2D adults, results of the focus groups with adults and adolescents (aged 14 to 17 years) with diabetes, and formation of communities of practice aimed to co-design the MOOCs. The literature review and survey results were reported in the final project report [33,34]. In this paper, we report the results of the focus groups and formation of communities of practice.

### Recruitment and Procedure

Participants were recruited from primary care centers, hospitals, and social networks following a snowball sampling approach [35]. There were no exclusion criteria. The confidentiality of patient personal data was guaranteed in accordance with the European Commission’s guidelines.

Three focus groups were held in Spain and Italy between March and April 2017 following a semistructured guide to qualitatively explore the dimensions of digital health literacy and complement the information from the survey. All discussions were audiorecorded.

**Figure 1:**
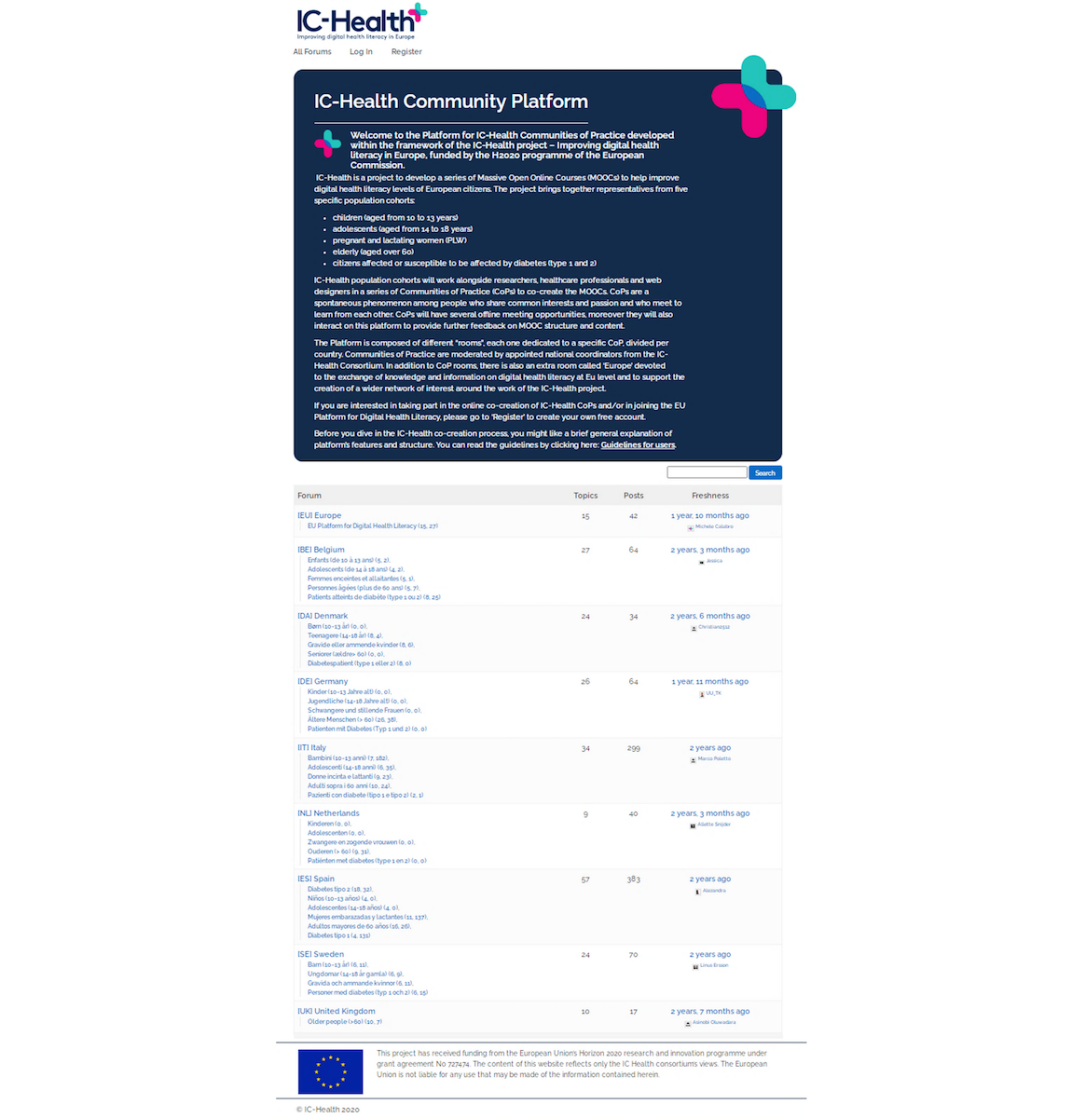
Screenshot of the platform for cocreation activities.

The cocreation process to develop the MOOCs was accomplished by creating communities of practice [36,37] independently by country and diabetes type. Each one comprised key stakeholders (people with T1D or T2D, endocrinologists, nurses, pediatric diabetologists, psychologists, and researchers) and was organized and coordinated by a project researcher through a closed Moodle learning management system platform (a screenshot of the platform is shown in Figure 1).

Within each community of practice, the cocreation process started with a face-to-face group session with the participants that lasted approximately 2 hours in each country. In these first sessions, the preliminary storyboard of each MOOC according to the dimensions of finding, understanding, appraisal, and applying health information was defined. Subsequently, participants continued to participate in the cocreation process through a web platform between October 2017 and April 2018. The community of practice coordinator shared the drafts developed for each skill weekly; requested feedback on the contents, format, and graphic materials; and promoted interaction between participants. Participant feedback on the content and design of each MOOC was considered for the pertinent modifications.

Finally, after the online participation, another round of face-to-face sessions was held. In these sessions, participants provided their final feedback on navigation, accessibility, content, and structure of the MOOCs, and quantitative measures were applied. Therefore, this pilot quantitative assessment was performed in the same sample that participated in the cocreation process. All sessions followed a semistructured guideline.

### Quantitative Measures

The following questionnaires were administered either in face-to-face sessions and on the Moodle platform:

- Acceptability of the MOOCs was assessed through a 14-item questionnaire (developed specifically for this project and based in previous related studies [38]) that evaluated ease of navigation, clarity of the objectives and language, appropriateness of learning activities, and other characteristics of the MOOCs (Multimedia Appendix 1).
- Experience during the cocreation process was assessed by means of 3 self-developed items rated on a 4-point Likert scale from 0 (totally disagree) to 4 (totally agree): (1) “Being part of the cocreation process made the MOOC content more relevant to my needs,” (2) “The cocreation process made me feel part of the project,” and (3) “Taking part in the different workshops has improved my knowledge about digital health literacy. This has increased my ability to take charge of my health” Multimedia Appendix 2).
- Self-perceived digital health literacy was assessed before and after the MOOCs development. We used 5 items from the eHealth Literacy Scale [39], 2 items from the eHealth Impact Questionnaire [40], and one item from the Health Literacy Questionnaire [41]. Items assessed 3 main skills required in digital health literacy (finding, understanding, and appraising information on the internet; Multimedia Appendix 3).

### Analysis

#### Qualitative Analysis

The focus groups were analyzed by means of a descriptive deductive content analysis [42]: (1) in-depth analysis of the audio-registration, (2) identification of relevant issues discussed, (3) codification of each relevant topic, (4) clustering of information obtained on each topic, (5) critical analysis and interpretation of information collected on each explored topic, (6) incorporation of the moderator and assistant observations, and (7) synthesis of results. The results of the focus groups were exploratory and informed the cocreation of semistructured guidelines in Spain and Italy.

#### Quantitative Analysis

Means and standard deviations were calculated for each item measuring acceptability, cocreation experience, and digital health literacy scales. Nonparametric analyses were used to compare results between countries (Mann-Whitney *U* test) in acceptability and experience items and within samples in digital health literacy (Wilcoxon signed-rank test) before and after the cocreation process.

## Results

### Focus Groups

A total of 8 Italian adolescents with T1D and 20 Spanish adults with T1D or T2D participated in the focus groups (Table 1).

The following main themes were identified: experiences, needs, expectations, and trust in the use of the internet as a source of information on health and illness issues (Table 2).

**Table 1:** Characteristics of the participants in focus groups (n=28).

Characteristics				Total diabetes participants (n=28)		Total T1D^a^ participants (n=18)		Total T2D^b^ participants (n=10)
**Country, n (%)**								
	Spain (adults)			20 (71)		10 (56)		10 (100)
	Italy (adolescents)			8 (29)		8 (44)		—^c^
**Age range (years)**								
	Spain (adults)			22-75		22-54		35-75
	Italy (adolescents)			14-17		14-17		—
**Gender, n (%)**								
	**Spain (adults)**							
		Female	11 (39)		5 (28)		6 (60)	
		Male	9 (32)		5 (28)		4 (40)	
	**Italy (adolescents)**							
		Female	3 (11)		3 (17)		—	
		Male	5 (18)		5 (28)		—	
**Educations, n (%)**								
	**Spain (adults)**							
		Primary education	1 (34)		1 (6)		—	
		Secondary school	3 (11)		—		3 (30)	
		Medium/high technical education	4 (14)		1 (6)		3 (30)	
		Undergraduate	5 (18)		2 (11)		3 (30)	
		University degree	7 (25)		6 (33)		1 (10)	
	**Italy (adolescents)**							
		High school	8 (29)		8 (44)		8 (29)	
**Civil status, n (%)**								
	**Spain (adults)**							
		Married/living with partner	7 (35)		4 (40)		3 (30)	
		Separated or divorced	5 (25)		1 (10)		4 (40)	
		Single	6 (30)		5 (50)		1 (10)	
		Widow	2 (10)		—		2 (20)	
**Employment status, n (%)**								
	**Spain (adults)**							
		Employed	5 (25)		4 (40)		1 (10)	
		Unemployed	5 (25)		3 (30)		2 (20)	
		Retired	8 (40)		1 (10)		7 (70)	
		Student	2 (10)		2 (20)		—	

^a^T1D: type 1 diabetes.

^b^T2D: type 2 diabetes.

^c^Not applicable.

**Table 2:** Themes and subthemes identified in the thematic analysis.

Themes	Subthemes	Example quote
Experience/general opinion using internet for health and illness issues	Personal experiences Level of satisfaction Use of this information	“I trust my doctor a lot, but I sometimes go into the internet to nose around.” “I read that a new resolutive treatment for diabetes was found but then going deeper in other websites I realized that the information was false.” “I used the web for a medical advice about diabetes (insulin question), but I didn’t find the specific answer and I had to call the hospital.”
Needs and expectations of the use of internet as source of information on health and illness issues	Informational needs Preferences relating display format	“It would be very interesting internet forums, for example, that we are all from here, or wherever, if we could all have a forum to share our experiences and encourage each other.” “I prefer websites because they are easier to use, you don’t need a smartphone, you don’t have to download anything, and it doesn’t take too much space in the memory of the device.”
Trust on internet as source of information on health and illness issues	Situations of NOT using Why you trust information Issues enhance or diminish level of trust	“On the internet, you can find everything but then you have to ask your medical doctor, especially for big issues or emergency.” “On the internet a lot of things can be dumped. I think you can trust the government websites; they should hang those reliable pages.” “Social media, such as Facebook, tends to produce a lot of false information; eg, they often claim a permanent cure for diabetes.”

The T2D group was older, which is related to the social distribution of this health problem. The T1D adult group was younger and used the internet more frequently. Adolescents with T1D used the internet every day. In general, all patients preferred images and videos with nontechnical language for better comprehension.

In the T2D group, internet use was variable. Almost all participants used the internet, but most of them stated they did not use it when related to health issues. Not all the participants were sure about how to establish trust in content found on the internet, and the internet was mainly considered a secondary health information source. We found 2 types of profiles of patients among the participants: those newly diagnosed patients who had very little information and those with a long-term diagnosis, more informed but with some myths and beliefs. Most of the participants demanded information about self-management in relation to eating (practical information about what to eat and how to find sugar level for different foods; see Multimedia Appendix 4 for illustrative quotes).

Most adults participants with T1D felt comfortable reading and using online health content and considered the internet a secondary source of information. Adults with T1D tended to seek practical information that helped them with everyday decision-making in their self-management. They demanded information on management of hypoglycemia, interaction between insulin intake and physical exercise and precise nutritional information (regarding food labels, ration calculation, adjusting insulin intake, and the sensibility insulin factor). The main worry in the group was avoiding hypoglycemia and its consequences (Multimedia Appendix 4).

Adolescents with T1D used the internet for searching for health-related information. They agreed that the internet has never or hardly ever been the only or first source of health-related information. Most adolescents with diabetes said they use the internet but they face difficulties in establishing what is fake or reliable. Most participants reported that they would use the internet only for minor problems, immediate questions, to verify consequences of diabetes bad metabolic control, to understand therapies different from insulin and new types of insulin, to talk with other diabetic patients, and get updates about new technology for diabetes. For emergency and major problems or health questions, they would not use the web because of the overwhelming amount of information. Most participants expressed they would like information about how to recognize symptoms and diabetes complications that is tailored to personal needs (Multimedia Appendix 4).

### Cocreation Process: Community of Practice and MOOCs Developed

**Figure 2:**
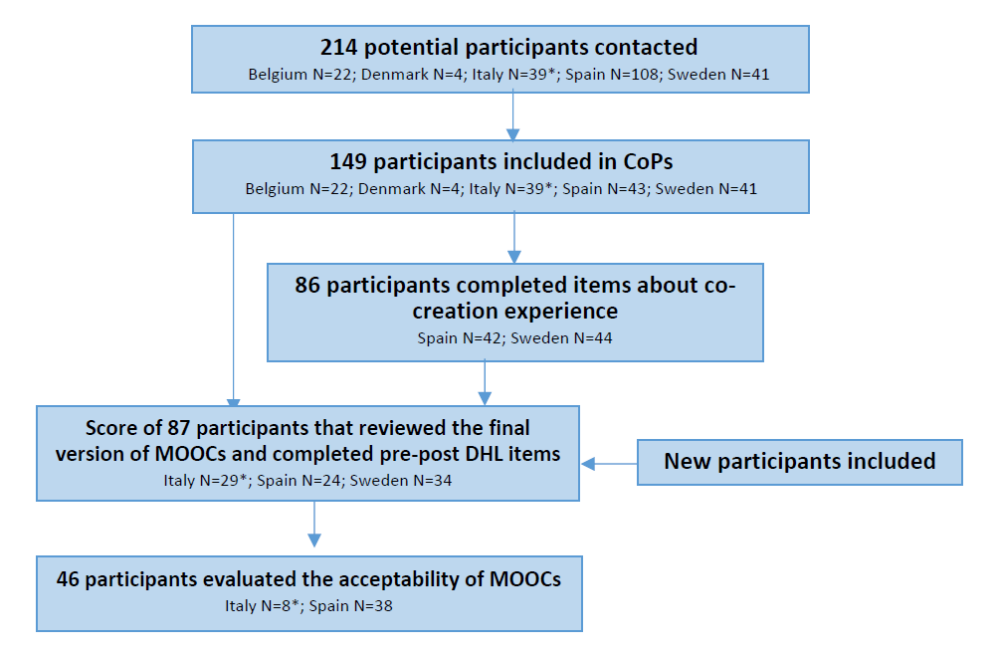
Flow of participants in the study. *Italian participants were children and adolescents (10-13 years).

A total of 214 people with diabetes were invited to participate in the communities of practice, of which 149 agreed to participate and attended the first face-to-face session; the diabetes cohort consisted of 39 Italian children and adolescents (aged 10 to 13 years) and 110 adults from Spain, Belgium, Denmark, and Sweden (Figure 2).

A total of 66.4% (73/110) of adult participants were female and 50.0% (55/110) had T2D. The most frequent age range was 40 to 59 years (58/110, 52.7%). Of the adult participants, 39.1% (43/110) had a high school diploma and 82.7% (91/110) used the internet daily. Of the participating children and adolescents, 67% (26/39) were female and 74.4% (29/39) used the internet once or twice a week.

In some cases with the T2D cohort and children and adolescents, the communities of practice coordinators taught basic digital skills before starting the actual project and cocreation. These participants had difficulty with computers in general and the communities of practice platform and Moodle registration specifically because they had not used laptops or computers frequently. As a result, they had more difficulties with basic actions, such as log-in or creating an account for the communities of practice platform or the MOOCs.

Italian children, adolescents, and their parents were more willing to participate in face-to-face meetings. They stated that sessions should have been organized closer in time, of longer duration, and less intensive in order to closely follow the discussions on MOOC development. We tried to motivate the younger participants asking them directly what they wanted to learn and how they wanted to be taught to then implement feedback.

A total of 9 self-administered MOOCs were developed on a Moodle platform (2 from Belgium, 1 from Denmark, 2 from Italy, 2 from Spain, and 2 from Sweden). Initially, the duration of each MOOC was estimated to be 15 minutes; at completion, however, MOOCs had an average duration of 60 to 90 minutes including materials and resources added by request of the participants. This supplementary material is not mandatory to achieve an effective knowledge of each skill, but it will help users expand the information presented if necessary.

The structure and format of the materials in each MOOC were adapted to the interests of the diabetes participants in each country, but all of them comprised 4 compulsory topics referring to subskills of digital health literacy: find, understand, appraise, and apply. In addition to the compulsory units, including an introductory unit with an overview of the MOOC and an introduction to digital health literacy was strongly recommended to national coordinators.

Units included texts, videos, images and infographics, and links to documents and shared documents. Videos were relevant existing ones or ones recently produced by the national coordinators from feedback received in their communities of practice. Self-produced videos were developed using Animaker (Animaker Inc) or Powtoon (Powtoon Ltd) tools. In the case of images, communities of practice expressed they preferred images to be embedded in the MOOCs, and infographics were developed by national coordinators from the feedback of communities of practice.

Assessment questions were included while progressing through the courses and after each unit, and a postassessment was also included at the end of the MOOCs. Moreover, for some MOOCs certificates of attendance were issued when learners completed the course and answered the questions associated with the evaluation and impact assessment. MOOCs are accessible from anywhere, at any time, and for many participants, since no contact with the trainers is necessary and the activities are asynchronous.

An updated version of the Spanish MOOCs can be found on the website of the University of La Laguna [43] (Multimedia Appendix 5 and Multimedia Appendix 6).

### Quantitative Outcomes

#### Acceptability of the MOOCs

Acceptability data were available for 46 participants (Multimedia Appendix 7). When totally agree and agree categories were combined, more than 90% of participants thought the language and objectives of the course were clear, contents were consistent with the objectives, learning activities were useful, and they would recommend the MOOC to other people.

A total of 89% (41/46) of participants stated that the duration of the course was appropriate and it had met their expectations while 72% (33/46) stated that navigation was easy and 76% (35/46) said the examples provided were of high or very high quality.

The scores were similar for the Spanish and Italian subsamples, except for the quality of the examples, which was perceived as higher in the Spanish subsample (*P*<.001).

#### Experience During the Cocreation Process

Data were available for 86 participants. The percentage of Spanish participants who agreed or totally agreed was 76% for the 3 items, whereas in Sweden it was 91%, 100%, and 86%, respectively (Table 3). Mean differences between the two countries were significant for the 2 former items (*P*=.008 and *P*=.004, Mann-Whitney *U* test).

**Table 3:** Results on items about the experience of cocreation of the massive open online courses (n=86).

Question	Spain (n=42)			Sweden (n=44)	
	Agree/totally agree, n (%)	Mean (SD)	Agree/totally agree, n (%)		Mean (SD)
1. Because I was part of the cocreation process, the MOOC content felt more relevant to my needs.	36 (76)	2.98 (0.71)	40 (91)		3.36 (0.65)^a^
2. The cocreation process made me feel I was part of the project.	36 (76)	3.07 (0.86)	44 (100)		3.54 (0.50)^a^
3. Taking part in the different workshops has improved my knowledge about digital health literacy. This has increased my ability to take charge of my health.	36 (76)	3.14 (0.72)	38 (86)		3.23 (0.68)

^a^*P*<.01 for the mean difference between countries (Mann-Whitney *U* test). Score ranges: 0 to 5.

#### Digital Health Literacy Scores

Baseline data were available for 87 participants. Because of absence of postevaluation data, 25.6% (10/39) of Italian adolescents were eliminated from the analysis; their baseline scores were lower than completers in finding (*P*=.048), understanding (*P*=.04), and appraising (*P*=.07; not shown in Table 4). The remaining participants showed a significant increase in the understanding (z=0.58, *P*=.002) and appraising (z=0.30, *P*=.03) scales. Table 4 shows the prescores and postscores on the digital health literacy dimensions.

In the Spanish and Swedish samples, 43% (18/42) of Spanish adults and 23% (10/44) of Swedish adults (23%) were excluded from analyses due to the absence of baseline data; their postscores did not significantly differ from those of analyzed participants in any dimension. The Spanish sample significantly improved in finding (z=0.46, *P*=.03) and appraising (z=0.45, *P*=.04).

Finally, Swedish participants, who showed higher scores at baseline than the other 2 samples, significantly improved in 3 scales, with mean increases of 0.70 (finding, *P*=.002), 0.75 (understanding, *P*=.001), and 0.73 (appraising, *P*=.001).

**Table 4:** Pre-post differences (Wilcoxon signed-rank test) in digital health literacy (n=87)^a^.

Digital health literacy skills	Italy (n=29)^b^, mean (SD)				Spain (n=24), mean (SD)				Sweden (n=34), mean (SD)		
	Pre	Post	z (*P* value)	Pre		Post	z (*P* value)	Pre		Post	z (*P* value)
Finding	2.21 (0.75)	2.48 (0.93)	–1.09 (.28)	2.01 (0.86)		2.47 (0.44)	–2.24 (.03)	2.20 (0.88)		2.90 (0.76)	–3.10 (.002)
Understanding	2.07 (0.75)	2.65 (0.57)	–3.09 (.002)	2.14 (0.95)		2.44 (0.59)	–1.22 (.22)	2.45 (1.07)		3.20 (0.58)	–3.23 (.001)
Appraising	2.16 (0.69)	2.46 (0.55)	–2.24 (.03)	1.93 (1.02)		2.38 (0.54)	–2.03 (.04)	2.56 (0.95)		3.29 (0.62)	–3.23 (.001)

^a^Higher score is better (range 0-4); 10 Italian, 18 Spanish, and 10 Swedish participants were excluded due to the absence of baseline (Spain and Sweden) or postassessment (Italy) data.

^b^Adolescents.

## Discussion

### Principal Findings

This study has demonstrated the feasibility of developing an online resource to improve the digital health literacy of diabetes patients in a cocreation process with the target audience from the initial moments of the development process. The cocreation experience was positively valued by the participants; they felt part of the project and were willing to share ideas and discuss with their peers. Acceptability of the final MOOCs was good. Most of the participants would recommend the MOOC to other people, highlighting as positive aspects the clarity of the language, coherence between the contents and objectives, and usefulness of the learning activities. In the 3 subsamples in which self-perceived digital health literacy was assessed (Italy, Spain, Sweden), significant pre-post improvements were observed in the appraising information scale and at least 1 out of the other 2 dimensions (ie, finding and understanding). However, these quantitative results are preliminary and exploratory, and they must be interpreted cautiously, since evaluation of the effectiveness of the MOOCs was not the main objective of the project.

Usability and easily of navigation is an essential factor for any MOOC to be accepted by the users to whom it is addressed. In the subsamples assessed, this feature was poorly valued by 9% (4/46) of participants, whereas 20% (9/46) were not sure. We observed more difficulties in T2D patients, which is not surprising since this group includes more senior patients who are less familiar with the use of new technologies [44]. Apart from teaching them basic digital skills, we tried to promote their involvement by actively asking them for advice and suggestions during the MOOC development, trying to increase their motivation, awareness, and interest around digital health literacy topics [45].

Many participants wanted more face-to-face sessions, which are more difficult to organization than online sessions and require a well-designed schedule that accommodates job and school calendars so face-to-face meetings can be possible.

Two of the most important lessons for a successful cocreation process that can be drawn from our experiences are the communities of practice coordinator must have the necessary skills to motivate users to actively participate in the community and interventions directed to people with T2D must consider the previous digital literacy level of the participants, since many may be elderly. Overall, participants felt part of the project, and they were willing to share ideas and discuss them with their peers.

### Strengths and Limitations

This study has several limitations. The focus groups were not originally part of the project and were held based on the subsequent initiative of the partners. Regarding the communities of practice, the risk of selection bias is present, since participants were not randomly recruited and the participation rate was low in the larger sample (Spain). Therefore, it is possible that the sample was not representative in terms of motivation or digital health literacy. Future studies should assure that people with low literacy levels are included in the cocreation process and evaluation of the MOOCs to avoid widening the digital divide. The results of the pilot quantitative analyses are subjected to several limitations and must be interpreted with caution. Acceptability and the change in self-perceived literacy were not assessed in a sample independent of the cocreation process. The scale used for digital health literacy was short and not psychometrically validated. Furthermore, we have not evaluated objective performance on digital health literacy, which is necessary to demonstrate the utility of the MOOCs for improving diabetes knowledge and self-management.

Developing initiatives to promote self-management as a strategy to empower patients is a practice increasingly implemented around the world [46]. Digital-based interventions are designed to extend accessibility and improve attractiveness for people with a wide range of health literacy levels [47]. Comparison and integration of valid information found in patients’ online searches with the information provided by their health care professionals can improve their knowledge and preferences related to treatment selection and use and decrease health risks due to poor understanding of online information or its reliability [48]. In the case of diabetes, although numerous interventions have been developed to improve health literacy and self-management, there is a large heterogeneity of intervention types and content and low completion rates, which produces mixed results [49-51]. The IC-Health project used a common methodology to develop a set of tools, in MOOC format, to promote digital health literacy of people with diabetes through materials accessible from anywhere, at any time, and for many participants, thus overcoming some barriers to the traditional education and training of this type of patients due to physical space limitations [52,53]. When cocreating MOOCs or any other e-learning content for people with diabetes, involvement of the target audience is recommended to maximize the likelihood that the final product is adapted to the needs and preferences of the end users [54-56].

### Conclusions

The results of the IC-Health project in people with diabetes show that MOOCs could be an accepted and effective way to improve the digital health literacy of diabetes patients and empower them to optimize their self-management. The cocreation experience in the development of MOOCs was positive for most of the participants. This methodology could reduce the limitations associated with low digital health literacy and other communication barriers in this population. More studies focusing on assessing the effectiveness and impact of the MOOCs on self-perceived and objective digital health literacy and health status of diabetes people are necessary.

## Appendix

Multimedia Appendix 1 Usability evaluation questionnaire.

Multimedia Appendix 2 Cocreation experience questionnaire.

Multimedia Appendix 3 Self-perceived digital health literacy questionnaire.

Multimedia Appendix 4 Illustrative quotes from focus groups.

Multimedia Appendix 5 Screenshot of updated version of the Spanish massive open online course: general appearance of the massive open online course for type 1 diabetes mellitus.

Multimedia Appendix 6 Screenshot of updated version of the Spanish massive open online course: example of the content of the unit on appraisal of the massive open online course for type 2 diabetes mellitus.

Multimedia Appendix 7 Acceptability of massive open online courses.

## Abbreviations

MOOC: massive open online course
T1D: type 1 diabetes
T2D: type 2 diabetes
