# Supplementary material for: Cocreation of Massive Open Online Courses to Improve Digital Health Literacy in Diabetes: Pilot Mixed Methods Study
Source: JMIR Diabetes. 2021 Dec 13;6(4):e30603. doi: 10.2196/30603 (PMC8713090; doi:10.2196/30603)
Supplement: Multimedia Appendix 1 [file diabetes_v6i4e30603_app1.pdf]

1. *The MOOC is easy to use/navigate and information was clearly organized.*
   ❑ Strongly Disagree ❑ Disagree ❑ Undecided ❑ Agree ❑ Strongly Agree

2. *The language on the MOOC was easy to understand.*
   ❑ Strongly Disagree ❑ Disagree ❑ Undecided ❑ Agree ❑ Strongly Agree

3. *This course has met my expectations.*
   ❑ Strongly Disagree ❑ Disagree ❑ Undecided ❑ Agree ❑ Strongly Agree

4. *The objectives of the course were made clear.*
   ❑ Strongly Disagree ❑ Disagree ❑ Undecided ❑ Agree ❑ Strongly Agree

5. *The course content was consistent with the course objectives.*
   ❑ Strongly Disagree ❑ Disagree ❑ Undecided ❑ Agree ❑ Strongly Agree

6. *The learning activities were useful to gain a clear understanding of the course content?*
   ❑ Strongly Disagree ❑ Disagree ❑ Undecided ❑ Agree ❑ Strongly Agree

7. *How would you rate the quality/usefulness of the examples provided in the course?*
   ❑ Very high quality ❑ High quality ❑ Undecided ❑ Low quality ❑ Very low quality

8. *The quizzes did appropriately test the material presented in the course.*
   ❑ Strongly Disagree ❑ Disagree ❑ Undecided ❑ Agree ❑ Strongly Agree

9. *Was the amount of time appropriate for the course content?*
   ❑ YES ❑ NO
   - *IF NO) Please state if duration was too short or too long.*
     ❑ Too short ❑ Too long

10. *How would you rate the overall design and aesthetics of the course content and materials?*
    ❑ Very high quality ❑ High quality ❑ Undecided ❑ Low quality ❑ Very low quality

11. *I would recommend this course to other people.*
    ❑ Strongly Disagree ❑ Disagree ❑ Undecided ❑ Agree ❑ Strongly Agree

12. *Please provide a short summary of the strengths and weaknesses of the course.*
13. *Open question. Please provide brief suggestions on how to improve the course.*
14. *Open question. What are the main points that you have learned through this course?*
