# Supplementary material for: Cocreation of Massive Open Online Courses to Improve Digital Health Literacy in Diabetes: Pilot Mixed Methods Study
Source: JMIR Diabetes. 2021 Dec 13;6(4):e30603. doi: 10.2196/30603 (PMC8713090; doi:10.2196/30603)
Supplement: Multimedia Appendix 2 [file diabetes_v6i4e30603_app2.pdf]

Experience during the co-creation sessions

1. *Being part of the co-creation process made the MOOC content more relevant to my needs*
   ❑ Strongly Disagree ❑ Disagree ❑ Undecided ❑ Agree ❑ Strongly Agree

2. *The co-creation process made me feel part of the project.*
   ❑ Strongly Disagree ❑ Disagree ❑ Undecided ❑ Agree ❑ Strongly Agree

3. *Open question. Which of the co-creation approaches did you think was most useful? Please provide a short (one or two sentences) description of the approaches that you found most useful.*
4. *Open question. Are there any issues or improvements you think are important? E.g. duration*

Effect of being part of the co-creation process on your ability to manage or take care of your own health.

5. *Taking part in the different workshop has improved my knowledge about digital health literacy. This has increased my ability to take charge of my health.*
   ❑ Strongly Disagree ❑ Disagree ❑ Undecided ❑ Agree ❑ Strongly Agree
