# Supplementary material for: Cocreation of Massive Open Online Courses to Improve Digital Health Literacy in Diabetes: Pilot Mixed Methods Study
Source: JMIR Diabetes. 2021 Dec 13;6(4):e30603. doi: 10.2196/30603 (PMC8713090; doi:10.2196/30603)
Supplement: Multimedia Appendix 3 [file diabetes_v6i4e30603_app3.pdf]

1. *I know how to find useful health resources on the Internet.*
   *❑ Strongly Disagree ❑ Disagree ❑ Undecided ❑ Agree ❑ Strongly Agree*

2. *I get nervous using the Internet to find information about my health.*
   *❑ Strongly Disagree ❑ Disagree ❑ Undecided ❑ Agree ❑ Strongly Agree*

3. *I know where to find useful health resources on the Internet.*
   *❑ Strongly Disagree ❑ Disagree ❑ Undecided ❑ Agree ❑ Strongly Agree*

4. *I know how to use the Internet to help me understand what I am not sure about my health.*
   *❑ Strongly Disagree ❑ Disagree ❑ Undecided ❑ Agree ❑ Strongly Agree*

5. *I can understand the health information I get from the Internet well enough to know what to do.*
   *❑ Strongly Disagree ❑ Disagree ❑ Undecided ❑ Agree ❑ Strongly Agree*

6. *I have the skills I need to evaluate the health resources I find on the Internet.*
   *❑ Strongly Disagree ❑ Disagree ❑ Undecided ❑ Agree ❑ Strongly Agree*

7. *I can differentiate high-quality health resources from low-quality health resources on the Internet.*
   *❑ Strongly Disagree ❑ Disagree ❑ Undecided ❑ Agree ❑ Strongly Agree*

8. *I feel confident in using information from the Internet to make health decisions*
   *❑ Strongly Disagree ❑ Disagree ❑ Undecided ❑ Agree ❑ Strongly Agree*
