# Supplementary material for: Cocreation of Massive Open Online Courses to Improve Digital Health Literacy in Diabetes: Pilot Mixed Methods Study
Source: JMIR Diabetes. 2021 Dec 13;6(4):e30603. doi: 10.2196/30603 (PMC8713090; doi:10.2196/30603)
Supplement: Multimedia Appendix 4 [file diabetes_v6i4e30603_app4.pdf]

Results Type I Diabetes (adults)

Experience/general opinion using internet for health and illness issues.

Participants used Internet every day. The use related to health issues was variable going from participants who did not searched for health content to does who looked as an everyday practice.

> *P8: No, I was going to say that I do not really seek for health information online.*
> *P1: Me neither.*
> *P8: I don't search. (...) Well, yes, sometimes I look for the carbohydrates on a recipe that I want to do.*
> *P5: I do, I look a lot, every... well almost every day.*

The majority of the participants felt comfortable reading and using on-line health content.

> *P3: And then really every doubt that comes to me. And it is true you have to know were to look .*

Participants' needs and expectations of the use of Internet as source of information on health and illness issues

They had less doubts and gaps of knowledges than participants on the Diabetes Type II FG. Nevertheless they still demand information on how to solve hypoglycaemia, how to act when they are sick, how insulin intake can relations with exercise, how to manage alcohol consumption.

> *P4: yes, all the information as hypoglycaemia symptoms, because, more or less, the symptoms, almost everybody knows them, o look, if I have an hyperglycaemia also, so, maybe another line hyperglycaemia, so something organised in different aspects of life*

Food and insulin intake were also a priority. Participants demanded more precise nutritional information, especially regarding food labels, ration calculation, adjusting insulin intake, the sensibility insulin factor. They are looking for diet flexibility. The main worry in the group was avoiding hypoglycaemias and their consequences.

> *P1: No, and the ignorance, because I didn't knew what a bolus was, I think it was sensibility insulin factor*

Participants also discussed the psychological aspects of Type I Diabetes. They would like more support on coping with this chronic condition, may be through forums dedicated to emotional aspects of the disease where patients can share experiences and mutual support.

> *P1: It would be very interesting Internet forums, for example, that we are all from here, as in the diabetic association, here in Gran Canaria, or wherever, if we could all have a forum to share our experiences and encourage each other.*

Participants' trust on internet as source of information on health and illness issues.

Participants were able to discuss about trust of the internet health content. Some sources, as personal social networks or unknown/distant universities were considered difficult to trust.

> *P2: Yes, in the Internet a lot of things can be dump, but know that for heart failure I have to look for the Spanish Cardiology Society (...)*
> *P3: And for diabetes, what search? What would I have to look? (...) P2: The Spanish Diabetes Society (...)*
> *Woman (unidentified): Yes, associations or public bodies...*
> *P8: I think you can trust the government web they should hang those reliable pages*

Results Type I Diabetes (adolescents)

Experience/general opinion using internet for health and illness issues.

Most adolescents with diabetes said they use the Internet, but they face difficulties to establish what is fake or reliable. Often, they find fake information about diabetes such as:

> *P1: "I read that a new resolutive treatment for diabetes was found but then going deeper in other websites I realized that the information was false".*

Most participants (5) said they would use the internet only for minor problems otherwise they ask directly their parents and doctor or diabetologist. For emergency and major problems/health questions they would not use the web because of overwhelming information.

> *"On the internet you can find everything but then you have to ask your medical doctor especially for big issues or emergency".*

Participants' needs and expectations of the use of Internet as source of information on health and illness issues

They said they would like to ask questions about different and unknown diseases related or not with diabetes, but with the virtual presence of a medical doctor.

> *P7: "It happens that information are referred to adults and when you read them you are frightened for something that doesn't matter. Information on the Internet is not specific for us".*

Participants' trust on internet as source of information on health and illness issues.

Most teenagers with diabetes recognize themselves as particularly well-educated people, therefore not necessarily representative of the general adolescents population because "usually our friends don't care about health".

> *P3: "Our peers don't pay attention at health issues because they don't have diabetes".*
> *P5: "We search for health information because we have a chronic disease. A person searches for something who has, for example a symptom or a disease. If a person is healthy, probably doesn't search anything".*

Results Type II Diabetes

Experience/general opinion using internet for health and illness issues.

In this group Internet use was very variable. Even if almost all participants (except 1) used the Internet, most of them stated they did not used them when related to health issues.

> *P4: I do not use the Internet at all. I trust much more my doctors, see, my nurses and all, I have never use the Internet to search anything related to health.*

Internet was mainly considered a secondary source of information. Participants preferred their doctors nurses, their family or patients associations as they are considered trusted sources of information.

> *P7: I trust my doctor a lot, but I sometimes go into the Internet to nose around*

Other participants had family members, as their parents, who had lived with diabetes for a long timeare were their main source of information. Other consulted sources of information were newspapersor magazine (specially does centred in diabetes), courses from the Spanish National Health Service.

M: And, you don't have doubts that you would like to seek in the Internet?
P4: No, not me, really. I have wonderful doctors, I do not have practically any doubts. I also have some magazines, a magazine that is called Diabetes, I read it a lot, and, really, there I get a lot of information about everything.

Participants' needs and expectations of the use of Internet as source of information on health and illness issues

Even though most of the participants demanded information about self-management in relation to food and eating. The main demand was for practical information for self-management, specially about what to eat and which is the level of sugar for the different foods. Some participants also asked for notions on how to manage hyperglycemia and hypoglycemia, sports or the consumption of alcohol beverages.

P9: I think something as really important, as that man was saying, if the web could include a good relation of foods that diabetics could eat depending on our type of diabetes.

Participants' trust on internet as source of information on health and illness issues.

Internet was mainly considered a secondary source of information. Participants preferred their doctors, nurses, their family, or patients' associations as they are considered trusted sources of information. Both family doctors and specialist doctors where specially mention as preferred sources of information. Nevertheless, trust issues were also risen regarding these healthcare professionals.

P8: But, actually, they study the same and they take care of being updated in the same thing, everyone is fine, everything.
P1: Yes but depending on how you worry about the patient P8: Exactly, that's another theme, ¿no? That depending...
