# Supplementary figures and images for: Cocreation of Massive Open Online Courses to Improve Digital Health Literacy in Diabetes: Pilot Mixed Methods Study
Source: JMIR Diabetes. 2021 Dec 13;6(4):e30603. doi: 10.2196/30603 (PMC8713090; doi:10.2196/30603)

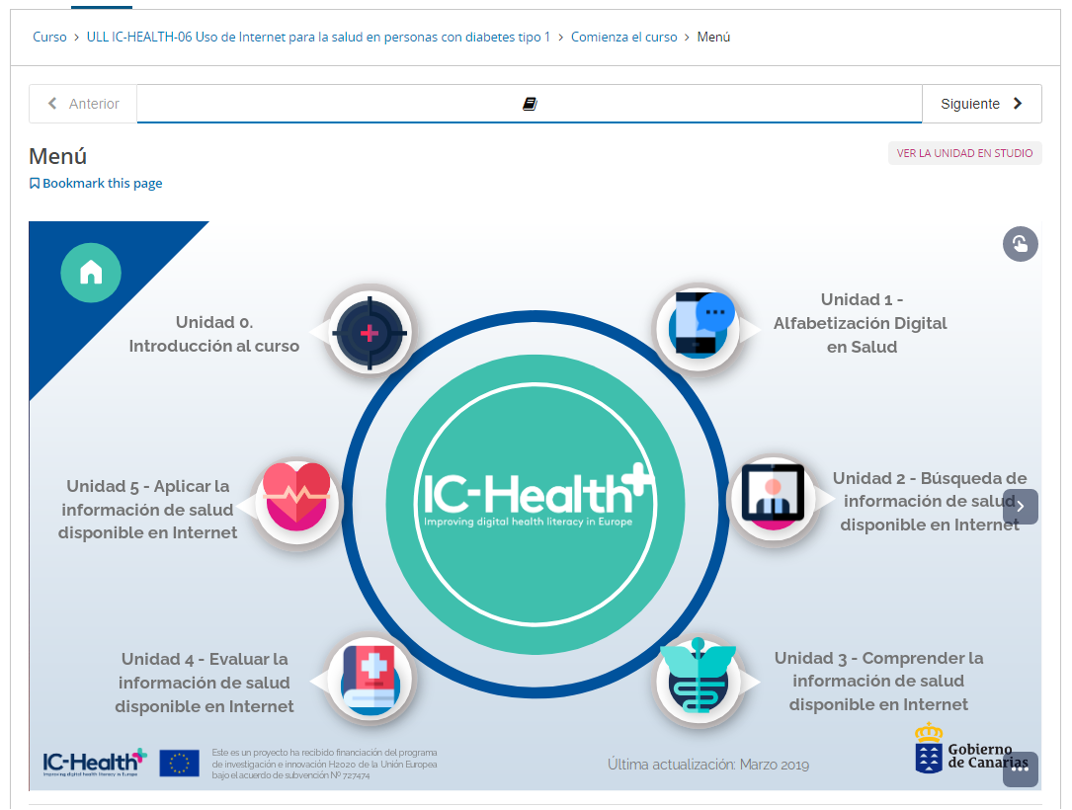

Supplement: Multimedia Appendix 5 [file diabetes_v6i4e30603_app5.png]

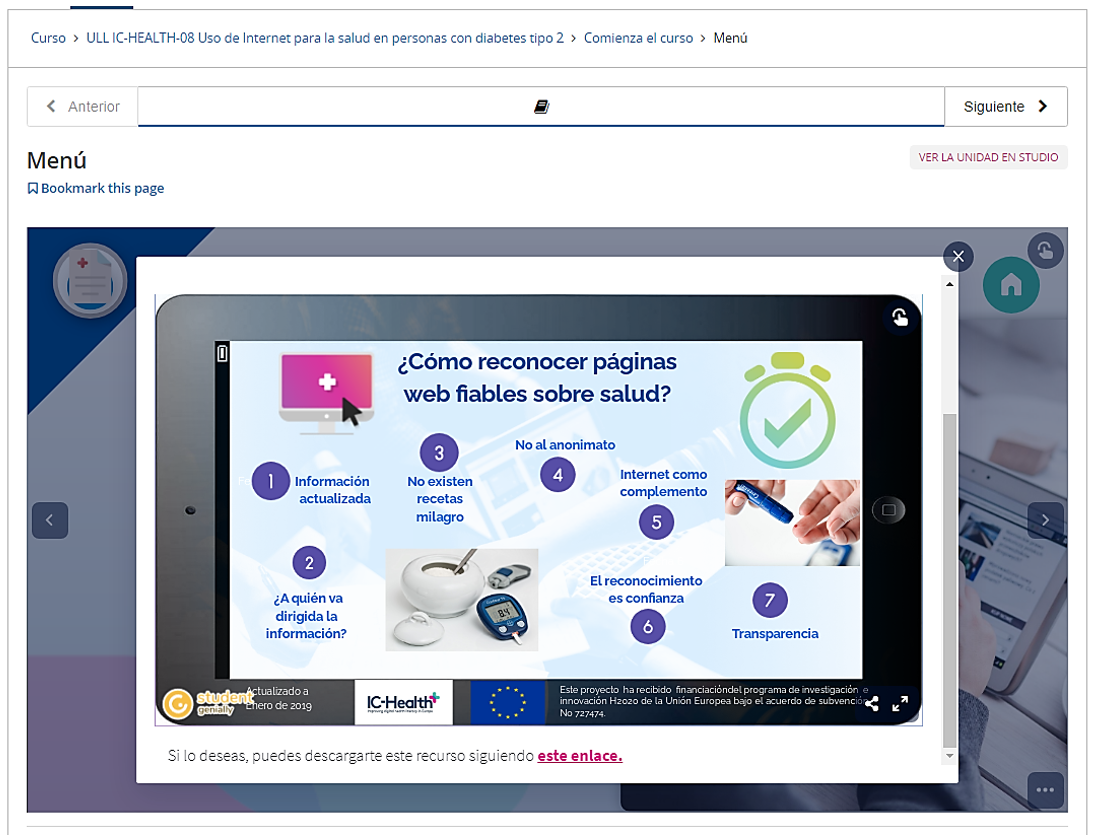

Supplement: Multimedia Appendix 6 [file diabetes_v6i4e30603_app6.png]
