# Supplementary material for: Cocreation of Massive Open Online Courses to Improve Digital Health Literacy in Diabetes: Pilot Mixed Methods Study
Source: JMIR Diabetes. 2021 Dec 13;6(4):e30603. doi: 10.2196/30603 (PMC8713090; doi:10.2196/30603)
Supplement: Multimedia Appendix 7 [file diabetes_v6i4e30603_app7.docx]

Multimedia Appendix 7. Acceptability of the MOOCs (n=46).

| Questions | Totally Agree,  n (%) | Agree,  n (%) | Not sure,  n (%) | Disagree,  n (%) | Totally disagree,  n (%) | Mean^a^ (sd) |
| --- | --- | --- | --- | --- | --- | --- |
| 1. The MOOC is easy to use/navigate and information was clearly organized | 11 (24) | 22 (48) | 9 (20) | 3 (6) | 1 (2) | 2.85 (0.94) |
| 1. The language on the MOOC was easy to understand | 14 (30) | 31 (68) | 1 (2) | 0 (0) | 0 (0) | 3.28 (0.50) |
| 1. The objectives of the course were made clear | 15 (33) | 29 (63) | 2 (4) | 0 (0) | 0 (0) | 3.28 (0.54) |
| 1. The course content was consistent with the course objectives | 13 (28) | 31 (67) | 1 (2) | 1 (2) | 0 (0) | 3.22 (0.59) |
| 1. The learning activities were useful to gain a clear understanding of the course content | 18 (39) | 26 (57) | 2 (4) | 0 (0) | 0 (0) | 3.35 (0.57) |
| 1. The quizzes did appropriately test the material presented in the course | 9 (20) | 22 (48) | 11 (24) | 4 (9) | 0 (0) | 2.78 (0.87) |
| 1. This course has met my expectations | 16 (35) | 25 (54) | 5 (11) | 0 (0) | 0 (0) | 3.24 (0.64) |
| 1. I would recommend this course to other people | 30 (65) | 13 (28) | 2 (4) | 1 (2) | 0 (0) | 3.56 (0.69) |
|  | Very high,  n (%) | High,  n (%) | Not sure,  n (%) | Low,  n (%) | Very low,  n (%) | Mean (sd) |
| 1. Quality of the overall design and aesthetics of the contents and materials | 6 (13) | 34 (74) | 3 (7) | 3 (7) | 0 (0) | 2.93 (0.68) |
| 1. Quality/usefulness of the examples provided in the course | 16 (35) | 19 (41) | 3 (7) | 7 (15) | 1 (2) | 2.91 (1.11) |
|  | Yes,  n (%) | | Too short,  n (%) | | Too long,  n (%) | |
| 1. Was the amount of time appropriate for the course content? | 41 (89) | | 4 (9) | | 2 (4) | |
| ^a^Higher scores indicate more positive rating (range 0-4). | | | | | | |
